# An overview of the Hadoop/MapReduce/HBase framework and its current applications in bioinformatics

**DOI:** 10.1186/1471-2105-11-S12-S1

**Published:** 2010-12-21

**Authors:** Ronald C Taylor

**Affiliations:** 1 Computational Biology and Bioinformatics Group, Pacific Northwest National Laboratory, Richland, Washington, 99352, USA

## Abstract

**Background:**

Bioinformatics researchers are now confronted with analysis of ultra large-scale data sets, a problem that will only increase at an alarming rate in coming years. Recent developments in open source software, that is, the Hadoop project and associated software, provide a foundation for scaling to petabyte scale data warehouses on Linux clusters, providing fault-tolerant parallelized analysis on such data using a programming style named MapReduce.

**Description:**

An overview is given of the current usage within the bioinformatics community of Hadoop, a top-level Apache Software Foundation project, and of associated open source software projects. The concepts behind Hadoop and the associated HBase project are defined, and current bioinformatics software that employ Hadoop is described. The focus is on next-generation sequencing, as the leading application area to date.

**Conclusions:**

Hadoop and the MapReduce programming paradigm already have a substantial base in the bioinformatics community, especially in the field of next-generation sequencing analysis, and such use is increasing. This is due to the cost-effectiveness of Hadoop-based analysis on commodity Linux clusters, and in the cloud via data upload to cloud vendors who have implemented Hadoop/HBase; and due to the effectiveness and ease-of-use of the MapReduce method in parallelization of many data analysis algorithms.

## Background on Hadoop/MapReduce/HBase

Due to new computational challenges (e.g., in next-generation sequencing [1,2] ), high performance computing (HPC) has become increasingly important in bioinformatics data analysis. HPC typically involves distribution of work across a cluster of machines which access a shared file system, hosted on a storage area network. Work parallelization has been implemented via such programming APIs as the Message Passing Interface (MPI) and, more recently, Hadoop’s MapReduce API. Another computer architecture/service model now being explored is cloud computing [3-5]. In brief, cloud computing equals HPC + web interface + ability to rapidly scale up and down for on-demand use. The server side is implemented in data centers operating on clusters, with remote clients uploading possibly massive data sets for analysis in the Hadoop framework or other parallelized environments operating in the data center.

### Hadoop

Hadoop [6-9] is a software framework that can be installed on a commodity Linux cluster to permit large scale distributed data analysis. No hardware modification is needed other than possible changes to meet minimum recommended RAM, disk space, etc. requirements per node (e.g., see Cloudera's guidelines [10]). The initial version of Hadoop was created in 2004 by Doug Cutting (and named after his son’s stuffed elephant). Hadoop became a top-level Apache Software Foundation project in January 2008. There have been many contributors, both academic and commercial (Yahoo being the largest such contributor), and Hadoop has a broad and rapidly growing user community [11,12].

Components - Hadoop provides the robust, fault-tolerant Hadoop Distributed File System (HDFS), inspired by Google's file system [13], as well as a Java-based API that allows parallel processing across the nodes of the cluster using the MapReduce paradigm. Use of code written in other languages, such as Python and C, is possible through Hadoop Streaming, a utility which allows users to create and run jobs with any executables as the mapper and/or the reducer. Also, Hadoop comes with Job and Task Trackers that keep track of the programs’ execution across the nodes of the cluster.

Data locality – Hadoop tries to automatically colocate the data with the computing node. That is, Hadoop schedules Map tasks close to the data on which they will work, with "close" meaning the same node or, at least, the same rack. This is a principal factor in Hadoop’s performance. In April 2008 a Hadoop program, running on 910-node cluster, broke a world record, sorting a terabyte of data in less than 3.5 minutes. Speed improvements have continued as Hadoop has matured [14].

Fault-tolerant, shared-nothing architecture - tasks must have no dependence on each other, with exception of mappers feeding into reducers under Hadoop control. Hadoop can detect task failure and restart programs on other healthy nodes. That is, node failures are handled automatically, with tasks restarted as needed. A single point of failure currently remains at the one name node for the HDFS file system.

Reliability – data is replicated across multiple nodes; RAID storage is not needed.

Programming support - unlike, for example, parallel programming using MPI, data flow is implicit and handled automatically; it does not need coding. For tasks fitting the MapReduce paradigm, Hadoop simplifies the development of large-scale, fault-tolerant, distributed applications on a cluster of (possibly heterogeneous) commodity machines.

MapReduce paradigm – Hadoop employs a Map/Reduce execution engine [15-17] to implement its fault-tolerant distributed computing system over the large data sets stored in the cluster's distributed file system. This MapReduce method has been popularized by use at Google, was recently patented by Google for use on clusters and licensed to Apache [18], and is now being further developed by an extensive community of researchers [19].

There are separate Map and Reduce steps, each step done in parallel, each operating on sets of key-value pairs. Thus, program execution is divided into a Map and a Reduce stage, separated by data transfer between nodes in the cluster. So we have this workflow: Input → Map() → Copy()/Sort() → Reduce() →Output. In the first stage, a node executes a Map function on a section of the input data. Map output is a set of records in the form of key-value pairs, stored on that node. The records for any given key – possibly spread across many nodes – are aggregated at the node running the Reducer for that key. This involves data transfer between machines. This second Reduce stage is blocked from progressing until all the data from the Map stage has been transferred to the appropriate machine. The Reduce stage produces another set of key-value pairs, as final output. This is a simple programming model, restricted to use of key-value pairs, but a surprising number of tasks and algorithms will fit into this framework. Also, while Hadoop is currently primarily used for batch analysis of very large data sets, nothing precludes use of Hadoop for computationally intensive analyses, e.g., the Mahout machine learning project described below.

HDFS file system – There are some drawbacks to HDFS use. HDFS handles continuous updates (write many) less well than a traditional relational database management system. Also, HDFS cannot be directly mounted onto the existing operating system. Hence getting data into and out of the HDFS file system can be awkward.

In addition to Hadoop itself, there are multiple open source projects built on top of Hadoop. Major projects are described such below.

### Hive

Hive [20] is a data warehouse framework built on top of Hadoop, developed at Facebook, used for ad hoc querying with an SQL type query language and also used for more complex analysis. Users define tables and columns. Data is loaded into and retrieved through these tables. Hive QL, a SQL-like query language, is used to create summaries, reports, analyses. Hive queries launch MapReduce jobs. Hive is designed for batch processing, not online transaction processing – unlike HBase (see below), Hive does not offer real-time queries.

### Pig

Pig [21] is a high-level data-flow language (Pig Latin) and execution framework whose compiler produces sequences of Map/Reduce programs for execution within Hadoop. Pig is designed for batch processing of data. Pig’s infrastructure layer consists of a compiler that turns (relatively short) Pig Latin programs into sequences of MapReduce programs. Pig is a Java client-side application, and users install locally – nothing is altered on the Hadoop cluster itself. Grunt is the Pig interactive shell.

### Mahout and other expansions to Hadoop programming capabilities

Hadoop is not just for large-scale data processing. Mahout [22] is an Apache project for building scalable machine learning libraries, with most algorithms built on top of Hadoop. Current algorithm focus areas of Mahout: clustering, classification, data mining (frequent itemset), and evolutionary programming. Obviously, the Mahout clustering and classifier algorithms have direct relevance in bioinformatics - for example, for clustering of large gene expression data sets, and as classifiers for biomarker identification. In regard to clustering, we may note that Hadoop MapReduce-based clustering work has also been explored by, among others, M. Ngazimbi (2009 M.S. thesis [23]) and by K. Heafield at Google (Hadoop design and k-Means clustering [24]). The many bioinformaticians that use R may be interested in the “R and Hadoop Integrated Processing Environment” (RHIPE), S. Guhi’s Java package [25] that integrates the R environment with Hadoop so that it is possible to code MapReduce algorithms in R. (Also note the IBM R-based Ricardo project [26]). For the growing community of Python users in bioinformatics, Pydoop [27], a Python MapReduce and HDFS API for Hadoop that allows complete MapReduce applications to be written in Python, is available. These are samplings from the large number of developers working on additional libraries for Hadoop. One last example in this limited space: the new programming language Clojure [28], which is predominantly a functional language, e.g., a dialect of Lisp that targets the Java Virtual Machine, has been given a library (author S. Sierra [29]) to aid in writing Hadoop jobs.

### Cascading

Cascading [30] is a project providing a programming API for defining and executing fault tolerant data processing workflows on a Hadoop cluster. Cascading is a thin, open source Java library that sits on top of the Hadoop MapReduce layer. Cascading provides a query processing API that allows programmers to operate at a higher level than MapReduce, and to more quickly assemble complex distributed processes, and schedule them based on dependencies.

### HBase

Lastly, an important Apache Hadoop-based project is HBase [31], which is modeled on Google's BigTable database [32]. HBase adds a distributed, fault-tolerant scalable database, built on top of the HDFS file system, with random real-time read/write access to data. Each HBase table is stored as a multidimensional sparse map, with rows and columns, each cell having a time stamp. A cell value at a given row and column is by uniquely identified by (Table, Row, Column-Family:Column, Timestamp) → Value. HBase has its own Java client API, and tables in HBase can be used both as an input source and as an output target for MapReduce jobs through TableInput/TableOutputFormat. There is no HBase single point of failure. HBase uses Zookeeper [33], another Hadoop subproject, for management of partial failures.

All table accesses are by the primary key. Secondary indices are possible through additional index tables; programmers need to denormalize and replicate. There is no SQL query language in base HBase. However, there is also a Hive/HBase integration project [34,35] that allows Hive QL statements access to HBase tables for both reading and inserting. Also, there is the independent HBql project (author P. Ambrose [36]) to add a dialect of SQL and JDBC bindings for HBase.

A table is made up of regions. Each region is defined by a startKey and EndKey, may live on a different node, and is made up of several HDFS files and blocks, each of which is replicated by Hadoop. Columns can be added on-the-fly to tables, with only the parent column families being fixed in a schema. Each cell is tagged by column family and column name, so programs can always identify what type of data item a given cell contains. In addition to being able to scale to petabyte size data sets, we may note the ease of integration of disparate data sources into a small number of HBase tables for building a data workspace, with different columns possibly defined (on-the-fly) for different rows in the same table. Such facility is also important. (See the biological integration discussion below.)

In addition to HBase, other scalable random access databases are now available. HadoopDB [37,38] is a hybrid of MapReduce and a standard relational db system. HadoopDB uses PostgreSQL for db layer (one PostgreSQL instance per data chunk per node), Hadoop for communication layer, and extended version of Hive for a translation layer. Also, there are non-Hadoop based scalable alternatives also based on the Google BigTable concept, such as Hypertable [39], and Cassandra [40]. And there are other so-called noSQL scalable dbs of possible interest: Project Voldemort, Dynamo (used for Amazon’s Simple Storage Service (S3)), and Tokyo Tyrant, among others. However, these non-Hadoop and non-BigTable database systems lie outside of our discussion here.

## Use of Hadoop and HBase in Bioinformatics

### Use in next-generation sequencing

The Cloudburst software [41] maps next-generation short read sequencing data to a reference genome for SNP discovery and genotyping. Cloudburst was created by Michael C. Schatz at the University of Maryland (UMD). Schatz’s Cloudburst paper [42], published in May 2009, put Hadoop “on the map” in bioinformatics. Following release of Cloudburst, Schatz and colleagues at UMD and at Johns Hopkins University (e.g., B. Langmead) have developed a suite of algorithms that employ Hadoop for analysis of next generation sequencing data:

1) Crossbow [43,44] uses Hadoop for its calculations for whole genome resequencing analysis and SNP genotyping from short reads.

2) Contrail [45] uses Hadoop for de novo assembly from short sequencing reads (without using a reference genome), scaling up de Brujin graph construction.

3) Myrna [46,47] uses Bowtie [48,49], another UMD tool for ultrafast short read alignment, and R/Bioconductor [50] for calculating differential gene expression from large RNA-seq data sets. When running on a cluster, Myrna uses Hadoop. Also, Myrna can be run in the cloud using Amazon Elastic MapReduce [51].

Cloud computing results - Amazon Elastic Compute Cloud (EC2) [52] and Amazon Elastic MapReduce are Web services that provide resizable compute capacity in the cloud. Among other batch processing software, they provide Hadoop [53] . Myrna was designed to function in Elastic MapReduce as well as on a local Hadoop-based cluster. Obviously, Langmead et al. believe that cloud computing is a worthwhile computing framework, and they report their results using such in [47]. Also, Schatz has tested Crossbow on EC2 and believes that running on EC2 can be quite cost effective [5]. (Note: non-commercial services such as the IBM/Google Cloud Computing Initiative [54] are also available to researchers.) Also, Indiana University (IU) researchers have performed comparisons [55] between MPI, Dryad (Microsoft [56,57]), Azure (Microsoft), and Hadoop MapReduce, measuring relative performance using three bioinformatics applications. This work was summarized by Judy Qui of IU at BOSC 2010 [58]. The flexibility of clouds and MapReduce come off quite well in the IU testing, suggesting “they will become preferred approaches”.

### Use in other bioinformatics domains

In addition to next-gen sequencing, Hadoop and HBase have been applied to other areas in bioinformatics. M. Gaggero and colleagues in the Distributed Computing Group at the Center for Advanced Studies, Research and Development in Sardinia, have reported on implementing BLAST and Gene Set Enrichment Analysis (GSEA) in Hadoop [59]. BLAST was implemented using a Python wrapper for the NCBI C++ Toolkit and Hadoop Streaming to build an executable mapper for BLAST. GSEA was implemented using rewritten functions in Python and used with Hadoop Streaming for the MapReduce version. They are now working on development of Biodoop [60], a suite of parallel bioinformatics applications based upon Hadoop, said suite consisting of three qualitatively different algorithms: BLAST, GSEA and GRAMMAR. They deem their results “very promising”, with MapReduce being a “versatile framework”.

In other work, Andrea Matsunaga and colleagues at the University of Florida have created CloudBLAST [61], a parallelized version of the NCBI BLAST2 algorithm (BLAST 2.2.18) using Hadoop. Their parallelization approach segmented the input sequences and ran multiple instances of the unmodified NCBI BLAST2 on each segment, using the Hadoop Streaming utility. Results across multiple input sets were compared against the publicly available version of mpiBLAST, a leading parallel version of BLAST. CloudBLAST exhibited better performance while also having advantages in simpler development and sustainability. Matsunaga et al. conclude that for applications that can fit into the MapReduce paradigm, use of Hadoop brings significant advantages in terms of management of failures, data, and jobs.

In other work, Hadoop has been used for multiple sequence alignment [62]. In regard to HBase use, Brian O’Connor of University of North Carolina at Chapel Hill recently described the use of HBase as a scalable backend for the SeqWare Query Engine [63] at the BOSC 2010 meeting. Recent work on the design of the Genome Analysis Toolkit at the Broad Institute has created a framework that supports MapReduce programming in bioinformatics [64,65]. Hadoop has also emerged as an enabling technology for large-scale graph processing, which is directly relevant to topological analysis of biological networks. Lin & Schatz have recently reported on improving the capabilities of Hadoop-based programs in this area [66].

As to future work not yet reported: starting in August 2010, A. Tiwari is maintaining a list of Hadoop/MapReduce applications in bioinformatics on his blog site [67].

### Use in scientific cloud computing, biological data integration and knowledgebase construction

The U.S. Department of Energy (DOE) is exploring scientific cloud computing in the Magellan project [67], a joint research effort of the National Energy Research Scientific Computing Center (NERSC), Lawrence Berkeley National Laboratory, and of the Leadership Computing Facility at Argonne National Laboratory (ANL). Hadoop and HBase have been installed on a cluster at NERSC (40 nodes reserved for Hadoop, soon to double), and studies have been run using Hadoop in Streaming mode for BLAST computations. NERSC is evaluating the use of solid state (flash) storage on the Hadoop nodes [68]. Also, the DOE Joint Genome Institute has performed contig extension work using Hadoop on the NERSC cluster. The Hadoop cluster at ANL, now undergoing testing, will be available for researchers in late 2010 [69,70]. Users interested in using clouds for their research may fill out the Magellan Cloud Computing statement of interest form [71].

At the Environmental Molecular Sciences Laboratory, a national user facility located at DOE's Pacific Northwest National Laboratory (PNNL), we wish to develop a scientific data management system that will scale into the petabyte range, that will accurately and reliably store data acquired from our various instruments, and that will store the output of analysis software and relevant metadata. As a pilot project for such an effort, work started in August 2010 on a prototype data repository, i.e., a workspace for integration of high-throughput transcriptomics and proteomics data. This database will have the capacity to store very large amounts of data from mass spectrometry-based proteomics experiments as well as from next-gen high throughput sequencing platforms. The author (RCT) is building the pilot database on a 25-node cluster using Hadoop and HBase as the framework. In addition to such data warehousing / data integration work, we may envisage using Hadoop and HBase for the design of large knowledgebases operating on a cluster across the distributed file system. The U.S. Dept. of Energy is funding work on construction of large biological knowledgebases [72], and Kandinsky, a 68-node, 1088-core Linux cluster (64 GB RAM, 8Tb disk per node) running Hadoop (Cloudera distribution, under CentOS 5) and HBase was set up in 2010 at Oak Ridge National Laboratory as an exploratory environment [73,74]. Cloudburst has been installed as a sample Hadoop-based application, and the cluster is open to use by researchers wishing to conduct preliminary work towards knowledgebase construction and towards support of grant proposals for such.

## Conclusions

Hadoop and its associated open source projects have a diverse and growing community in bioinformatics of both users and developers, as can be seen from the large number of projects described above. A concluding point, extracted from preliminary work for the Hadoop/HBase based PNNL project, follows Dean & Ghemawat [15]. That is, for much bioinformatics work not only is the scalability permitted by Hadoop and HBase important, but also of consequence is the ease of integrating and analyzing various large, disparate data sources into one data warehouse under Hadoop, in relatively few HBase tables.

## Abbreviations

AWS: Amazon Web Services; API: application programming interface; BLAST: Basic Local Alignment Search Tool; BOSC 2010: Bioinformatics Open Source Conference, July 2010; DOE: U.S. Dept. of Energy; EC2: Elastic Compute Cloud; GSEA: Gene Set Enrichment Analysis; GB: gigabytes; HPC: High performance computing; HDFS: Hadoop Distributed File System; IU: Indiana University; JDBC: Java DataBase Connectivity; MPI: Message-Passing Interface standard for programming parallel computers; NCBI: National Center for Biotechnology Information; PNNL: Pacific Northwest National Laboratory, U.S. Dept. of Energy; S3: Simple Storage Service

## Competing interests

The author has no competing interests.

## Authors' contributions

RCT was sole author.
